# Neurodegenerative Disease: From Molecular Basis to Therapy, 2nd Edition

**DOI:** 10.3390/ijms26051929

**Published:** 2025-02-24

**Authors:** Claudia Ricci

**Affiliations:** Department of Medical, Surgical and Neurological Sciences, University of Siena, 53100 Siena, Italy; claudia.ricci@unisi.it

Neurodegenerative diseases, characterised by the progressive degeneration of neurons, are a heterogeneous group of largely age-related disorders that affect millions of people worldwide. Age is the single most important risk factor for the development of all neurodegenerative diseases, but genetic and environmental factors can also enhance the risk. The prevalence of these diseases is increasing with increasing life expectancy, resulting in a growing socio-economic burden associated with neurodegenerative diseases. Current treatments are mostly symptomatic, without addressing the underlying cause of the disease, and have little or no effect on disease progression. Despite their different manifestations, some mechanisms, such as the presence of misfolded protein aggregates and abnormal protein accumulation, are common to several neurodegenerative diseases. On the other hand, in several cases, it is becoming clear that the same disease can be caused by different factors in different people, making a precision medicine approach necessary. Understanding these mechanisms is fundamental to the development of future effective therapies. While significant progress has been made in recent years in elucidating critical mechanisms underlying the pathogenesis of neurodegenerative diseases, this progress is only beginning to be effectively translated into clinical practice.

The aim of this Special Issue is to provide an up-to-date overview of progress in neurodegenerative disease research, from understanding the molecular basis to developing new therapies. Although the challenge remains daunting, there is some evidence to suggest that we are on the right track to identifying effective therapies, also in the context of a precision medicine approach.

A large proportion of the papers in this Special Issue focus on therapeutic approaches to Alzheimer’s disease (AD), both as a review of previously published studies and as future perspectives. In their review, Kiss and colleagues [1] provide an up-to-date overview of the experimental evidence documenting the neuroprotective activities of artemisinins, highlighting the potential of these drugs for the treatment of Alzheimer’s disease in humans and suggesting their consideration for carefully designed clinical trials. Artemisinin and its derivatives are plant-based drugs successfully used to treat malaria caused by Plasmodium parasites [2]. The authors describe the relatively large number of well-conducted studies, indicating the beneficial effects of these drugs in the preclinical setting of Alzheimer’s disease, showing improved pathological features and pointing to multiple disease causes that can be modulated to enhance cognitive function. At the same time, they underline that the lack of concerted validation of the doses and specific efficacy of each artemisinin compound, and comparisons of their relative efficacy in different animal models makes direct translation into clinical trials difficult. The authors point out that questions about dosing regimens, long-term safety and potential interactions with existing drugs, as well as toxicities that may be associated with treatment in Alzheimer’s patients can only be adequately answered by well-conducted clinical trials. 

Rakovskaya and colleagues [3] focused on one of the key pathogenic events associated with Alzheimer’s disease, the dysregulation of neuronal calcium (Ca^2+^). Pharmacological agents capable of stabilising neuronal Ca^2+^ signalling have been identified as potential disease-modifying agents in Alzheimer’s disease. The authors evaluated the effects of a set of novel positive allosteric regulators (PAMs) of the sarco/endoplasmic reticulum Ca^2+^ ATPase (SERCA) pump agents on the HEK293T cell line and 5XFAD transgenic mice modelling Alzheimer’s disease. Several SERCA PAM compounds showed neuroprotective properties, confirming their potential role as a therapeutic target for the treatment of AD, with the compound NDC-9009 showing the best results, offering promising prospects for the development of disease-modifying agents for Alzheimer’s disease.

5XFAD transgenic mice were also used as an AD model in the study performed by Park and colleagues [4]. They investigated the potential of adoptive regulatory T-cell (Treg) therapy for the treatment of Alzheimer’s disease. The authors developed a Treg preparation protocol to facilitate the clinical application of this therapy and evaluated the therapeutic effects in 5XFAD mice. In addition to improving cognitive function, Aβ-specific Tregs reduced Aβ and pTAU accumulation in the hippocampus of 5XFAD mice and inhibited microglial neuroinflammation. These effects were observed at very low doses. The results of this study suggest that Aβ-specific Tregs can attenuate AD pathology in 5XFAD mice, opening new perspectives for targeted immunotherapy of AD.

Kim and colleagues [5] propose a novel approach to characterise novel mediators in Alzheimer’s disease pathology. Their study highlights the importance of understanding the impact of exosomes on neural networks to improve our understanding of intracerebral neuronal communication and its impact on neurological disorders such as AD. The exosomes, derived from the nasal lavage fluid of 5XFAD mice, were studied using a high-density multielectrode array (HD-MEA) system, a novel technology that allows simultaneous recordings from thousands of neurons in primary cortical neuron cultures and organotypic hippocampal slices. The results showed increased neuronal firing rates and disoriented connectivity, reflecting the effects of pathological amyloid-beta oligomer treatment. Abnormal rhythmicity and increased current source density were also seen in local field potentials in exosome-treated hippocampal brain slices. This groundbreaking research is only a first exploration, but it shows the potential of exosomes to modulate neural networks and highlights the importance of understanding this modulation during the progression of Alzheimer’s disease.

The availability of appropriate animal models is a key issue in the development of new treatments for Alzheimer’s disease. In their perspective article [6], Volloch and Rits-Volloch provide an exhaustive review of the animal models currently used in AD research and point out their limitations. First, they are unable to develop the full spectrum of Alzheimer’s pathology. Secondly, they are very responsive to drugs that are completely ineffective in the treatment of symptomatic Alzheimer’s disease. This leads the authors to conclude that both the transgenic animal models and the drugs closely reflect the theory that guided their design, and that both fail because of the inadequacy of the underlying theory. They introduce a new, all-encompassing theory of conventional AD—the ACH2.0. The theory is also well described in the second paper of Volloch and Rits-Volloch present in this Special Issue [7]. In brief, it proposes that AD is a two-stage disease. Both stages are driven by intraneuronal (rather than extracellular) Aβ (iAβ), albeit from two distinct origins. The first asymptomatic stage is the accumulation of iAβ derived from the Aβ protein precursor (AβPP) over decades until a critical threshold is reached. This triggers the activation of the self-sustaining AβPP-independent iAβ production pathway and the onset of the second, symptomatic stage of AD. Importantly, AβPP-independent Aβ production is maintained intraneuronally. It drives AD pathology and perpetuates pathway function. In light of this theory, the authors conclude that current animal models are inadequate because they do not take into account the intraneuronal AβPP-independent iAβ production pathway and that this mechanism must be incorporated into any successful AD model that faithfully mimics the disease. The authors also propose principles for the design of novel transgenic animal models of the disease and describe the molecular details of their construction [6]. At the same time, the ACH2.0 could also guide a next-generation therapeutic strategy for the treatment and prevention of both AD, as described in the second paper [7]. It should lead to the depletion of iAβ via its transient, short-lived, targeted degradation. The authors also propose two plausible ACH2.0-based drugs, activators of the physiologically occurring intra-iAβ-cleaving capabilities of BACE1 and/or BACE2, as potential novel treatments.

The study by Butler and colleagues [8] investigated the dysregulation of (AD)-associated protein expression in polycystic ovary syndrome (PCOS). The authors measured the plasma levels of amyloid-associated proteins (Amyloid-precursor protein (APP), alpha-synuclein (SNCA), amyloid P-component (APCS), Pappalysin (PAPPA), Microtubule-associated protein tau (MAPT), apolipoprotein E (apoE), apoE2, apoE3, apoE4, Serum amyloid A (SAA), Noggin (NOG) and apoA1 in weight and aged-matched non-obese PCOS and control women. The dementia-related proteins fibronectin (FN), FN1.3 and FN1.4; Von Willebrand factor (VWF); and extracellular matrix protein 1 (ECM1) were also measured. Only APCS differed between groups, being elevated in non-obese PCOS women compared to non-obese controls. This differed markedly from the elevated APP, APCS, ApoE, FN, FN1.3, FN1.4 and VWF previously reported in obese women with PCOS [9]. Non-obese PCOS subjects had a lower AD-associated protein pattern risk profile than obese PCOS women and were more similar to non-obese controls. This suggests that maintaining optimal body weight may be fundamental to reducing the long-term risk of AD in women with PCOS.

Parkinson’s disease (PD) is another major focus of this Special Issue. Again, particular attention is paid to understanding the molecular mechanisms responsible for the disease and identifying future therapeutic targets. In their review, Battis and colleagues [10] describe the bidirectional interaction of α-synuclein with lipids and how its alterations may be related to the pathogenesis of Parkinson’s disease. The authors provide an accurate description of lipids and lipid metabolism in the central nervous system, describe their alteration in PD, and focus on the interactions between lipids and α-synuclein in physiological and pathological conditions. Finally, they propose several therapeutic approaches based on strategies to modulate the lipid-α-synuclein interaction. 

It is known that the accumulation of misfolded and aggregated α-synuclein can induce ER stress and unfolded protein response (UPR), leading to apoptotic cell death in Parkinson’s disease (PD) patients. A key role in the regulation of the UPR is played by glucose-regulated protein 78 (GRP78), the major ER chaperone [11]. In rat models of α-synuclein pathology, its overexpression can modulate the UPR, block apoptosis and promote survival of nigral dopamine neurons. Pazi and colleagues [12] investigated the therapeutic potential of intranasal exogenous GRP78 to prevent or slow PD-like neurodegeneration in a rat model. The intranasally administered GRP78 was rapidly internalised by neurons and microglia in the substantia nigra pars compacta and other affected regions of the brain and prevented the development of the neurodegenerative process in the nigrostriatal system. GRP78 treatment significantly reversed the abnormal accumulation of phosphorylated pS129-α-synuclein and activation of the pro-apoptotic pathway of the UPR. In addition, exogenous GRP78 inhibited both microglial activation and pro-inflammatory cytokine production. In light of these findings, the authors suggest that exogenous GRP78 may have neuroprotective and anti-inflammatory effects and may be an effective therapeutic agent for PD and other synucleinopathies.

Salemi and colleagues [13] carried out a transcriptome analysis of post mortem mRNA extracted from the substantia nigra of both PD patients and healthy controls. Using an RNA sequencing approach, they identified 33 mRNAs that were significantly up-regulated and 59 mRNAs that were down-regulated in PD compared to controls. An examination of statistically significant pathways using KEGG and GO enrichment analyses revealed the involvement of several signalling pathways including cardiac muscle contraction, GABAergic synapse, autophagy, and Fc gamma receptor-mediated phagocytosis. These results show that genes that are conventionally associated with electrical conduction mechanisms in cardiac muscle may also play a role in the brain, suggesting new pathophysiological mechanisms that underlie Parkinson’s disease. This knowledge could improve our understanding of Parkinson’s disease and contribute to the development of future targeted therapies.

Other papers in this Special Issue focus on the understanding of the general mechanisms underlying neurodegeneration, using animal models of specific conditions. Druga and colleagues [14] address the neurodegeneration associated with epilepsy using LiCl/pilocarpine to induce status epilepticus (SE) in rat pups. This is a widely accepted model of temporal lobe epilepsy that causes spontaneous recurrent seizures, cognitive and behavioural deficits, and extensive brain damage. The authors analysed the location of degenerating neurons in the dorsal (insular) claustrum (DCL and VCL) and the dorsal, intermediate and ventral endopiriform nucleus (DEn, IEn and VEn) after induction of SE at different postnatal days. This study showed that status epilepticus induced in the early stages of life causes neurodegeneration in the claustral complex. Age at SE induction and time intervals after SE were highly related to the extent and distribution of degenerating neurons. The severity of the damage increased with age at the time of SE and reached a peak at 24 h after SE. In the DCL, degenerating neurons predominated in the zone close to the medial and dorsal margins. Little degeneration was observed in the VCl, suggesting a protective role against SE-induced damage. 

Neuroinflammation and immune activation are widely accepted as significant contributing factors to the pathophysiology of neurodegenerative disease. Aries and colleagues [15] used low-level chronic peripheral LPS to induce neutrophil activation in the periphery and brain as a model to understand the role of inflammation in brain diseases. Subclinical levels of LPS were injected intraperitoneally into mice to study its effect on neutrophil numbers and activation. Since neutrophil activation in the periphery was higher in LPS-injected mice than in saline-injected mice after 4 weeks, but not after 8 weeks of injections, the time for brain examination was set at 4 weeks. Within this window, chronic LPS injections increased neutrophil activation in the periphery and brain of mice. These results indicate that subclinical levels of peripheral LPS induce neutrophil activation in the periphery and brain and define the experimental parameters. This model could be used for understanding how neutrophils may be mediators of the periphery–brain axis of inflammation in neurodegenerative or neuroinflammatory diseases.

Neurodegeneration may also involve progressive and relentless tissue loss in areas connected to an initial infarct-damaged zone, as in the case of stroke-related secondary neurodegeneration. Focusing on stroke, Hamblin and colleagues [16] examined the effects of MMP-3 genetic knockout (MMP-3 KO) on infarct volume and gene expression in the brains of mice subjected to middle cerebral artery occlusion followed by reperfusion. An RNA-seq analysis revealed a significant downregulation of gene expression signatures for neuroinflammation, endothelial and epithelial–mesenchymal transition, integrin cell surface signalling and apoptosis in the stroke brains of MMP-3 KO mice compared to MMP-3 wild-type controls, with a greater extent in females. Based on these results, the authors suggest that MMP-3 is a promising therapeutic target for improving stroke outcome because it affects multiple cellular pathways after stroke.

Given the evidence that increasing histamine levels has beneficial effects on memory [17] and that dihydroergotamine (DHE), an FDA-approved drug for the treatment of migraine, inhibits histamine N-methyltransferase (HNMT), the enzyme responsible for inactivating histamine in the brain [18], Hernández-Rodríguez and colleagues [19] evaluated the effect of DHE on histamine levels in the hippocampus and its effects on memory using a scopolamine-induced amnesia mouse model. Their results showed that DHE improves memory in the scopolamine-induced amnesia model by increasing histamine levels in the hippocampus through its activity as an HNMT inhibitor. As increasing histamine levels in the brain has been proposed as a promising approach to treating neurological disorders that cause memory impairment, these results may open up new therapeutic perspectives.

In their review, Ruqa and colleagues focus on the terminal nerve, a highly conserved and versatile nerve that is located just above the olfactory bulbs in humans and in several other species of vertebrates [20]. The authors summarise the main findings on the terminal nerve in order to clarify its anatomy and the various functions attributed to it, and to better interpret its possible involvement in pathological processes. They point out that the terminal nerve has been well studied in various animal species, but only a few studies have been carried out in humans. Therefore, its function remains unknown. The authors review studies suggesting a role in olfaction due to its proximity to the olfactory nerve. Others suggest a role in reproduction and sexual behaviour. It is thought to be involved in the unconscious perception of specific odours that influence the autonomic and reproductive hormone systems via the hypothalamic–pituitary–gonadal axis. Finally, a section of the review is devoted to the potential role of the terminal nerve in diseases such as Kallmann syndrome (a genetic form of hypogonadotropic hypogonadism) and COVID-19. 

Last but not least, the review by Cantara and colleagues [21] offers real hope for the advancement of therapeutic interventions in neurodegenerative disorders, addressing motor neuron diseases, namely spinal muscular atrophy (SMA), amyotrophic lateral sclerosis (ALS) and spinal bulbar muscular atrophy (SBMA). The authors reviewed the use of antisense oligonucleotides (ASOs), short oligodeoxynucleotides designed to bind to specific regions of target mRNA, as therapeutic agents in these diseases. This approach has led to major successes, such as the ASO known as nusinersen, the first effective treatment for SMA that can improve symptoms and slow disease progression. Another success is tofersen, an ASO designed to treat ALS patients with *SOD1* gene mutations. Both ASOs have received FDA and EMA approval and represent a milestone in the treatment of two diseases previously thought to be incurable. On the other hand, ASO treatment in ALS patients carrying the *C9orf72* gene mutation has failed to improve disease progression. The authors highlight the successes, failures, strengths and limitations of current ASO research and suggest approaches that could lead to more effective treatments.

In summary, this Special Issue addresses the problem of treating neurodegenerative diseases from a variety of perspectives, from basic research into the molecular mechanisms responsible for a disease to reviews of the most advanced therapies available. This topic can provide exciting insights into new therapeutic approaches for various neurodegenerative disorders, expanding our knowledge of the biological basis and clinical research, as well as new challenges and future perspectives in neurodegeneration.
